# On Extract of Indian Hemp

**Published:** 1847-01

**Authors:** Andrew Robertson

**Affiliations:** Professor of Chemistry to the Medical College, Calcutta


TRANSACTIONS OF THE PHARMACEUTICAL SOCIETY.

*On Extract of Indian Hemp.* By ANDREW ROBERTSON, Esq., Professor of Chemistry to the Medical College, Calcutta.—A number of pounds of the extract of hemp were prepared by me—I think upwards of thirty in all—for the purpose of having its medical properties fully tested by European medical men. A quantity went to Paris, another to Berlin, another to London, sent by different parties, and for my share of the matter I sent four pounds of it to Scotland, part of which went to you. I do not care about making more of it, as its preparation is most tedious and troublesome, in which I was tormented by the excise regulations of the country, for both the plant and the spirits used are the subject of heavy duties and stringent precautions, and the cost price of the extract to me, counting nothing for trouble, was fully fifteen shillings per lb. Dr. O'Shaughnessy made his extract with alcohol, in a Papin's digester, at a heat above the boiling point of alcohol—the extract so obtained is brown; mine is of a deep green, and gives a grass-green tincture to alcohol, and has six times the activity of the brown, as ascertained by trial on hospital patients. If a speedy effect is desired it is given as a tincture; if a deferred and protracted, as a pill.

As the process by which it was prepared is an idea of my own, since copied by others, and which probably may be claimed by them afterwards, I may mention it to you. It is a variation of the process of percolation, alcohol in vapour being the agent. A still was charged with strong spirits, and its nose introduced into the side of a cask in which the plant was pushed.

The vapour of the alcohol, and alcohol at a boiling heat thus acted on the plant, instead of cold alcohol in the usual mode of percolation. First issued a thin tarry matter containing much resin latterly, a brown liquor containing little resin but much extractive. At this point water was substituted for the spirit in the still, and as much as possible of the spirit retained by the plant thus expelled from it. From the bottom of the cask a pipe led to a common condensing worm. Part of the alcohol was recovered from the fluid by distillation, the rest dissipated by evaporation in Wedgewood ware on a sand-bath, not exceeding the temperature of 150 deg. Fahr. One hundred weight of the plant was used at one operation, and about eight pounds of extract obtained. The operation was conducted so slowly as in all its stages to last a fortnight.

The extract of hemp has been long known in the East, in a most widely extended range of countries, under the names of Gunjah, Churrus, Hashish, Beng or Bang, the emerald cup of Haider, &c., and under every name renowned for its exciting and narcotic qualities. It is used by the natives here in the same way as opium is by the Chinese, and on that account is the object of fiscal regulations and duties. It is known throughout all India, Arabia, Syria, and Eygpt. You will find it in the *Arabian Nights*, translated by Lane, under the name of Beng, as the narcotic used by Haroun Alraschid, and others. There cannot, therefore, be a doubt that it is a drug nearly as active as opium.

The inactivity of the drug, therefore, prepared in Britain, I can attribute only to faulty preparation and overheating, or to its being made from old and decayed plants. The good plant is of a greenish brown, the heads loaded with a sticky resin; the bad is palish-brown and does not adhere to the fingers. The good extract gives a grass-green tincture, the bad a brownish. My extract was made from dried plants of good quality, as it cannot be readily obtained fresh in Calcutta.

Mr. Fordred stated, that it had recently come to his knowledge that some of the extract, sold in London as extract of Indian hemp, was made from the plant grown in the neighborhood of London, and he believed possessed but little, if any, of the narcotic properties of the Indian plant. The extract made from the hemp (*Cannabis sativa*) grown at Mitcham, was of a green colour, and being apparently an aqueous extract contained but little resin, while that prepared from the plant grown in India contained a large proportion of resin. He thought it important, as many medical practitioners in different parts of the country were trying the efficacy of this remedy, that they should be cautioned to be particular in obtaining the extract of the *Indian* hemp.

Mr. Redwood said, that much of the extract made from the hemp plant imported from India, as well as the extract which had been imported ready made, was found to possess but little narcotic power when tried in this country; certainly they had not realized the expectations which were formed from the accounts of its action given by medical men in India. Dr. O'Shaughnessy, when last in this country, had admitted that the extract, even some that he had brought from India himself, had failed to produce the effects he anticipated, when tried in our hospitals; and he had undertaken, on his return to India, to have some extract very carefully prepared, and sent over to this country. Mr. Squire had received a quantity of this extract, and he presumed it was that alluded to in the paper just read, as having been made by Mr. Robertson.

Mr. Bartlett had witnessed the effect of a very small dose of extract of Indian hemp, obtained from Mr. Squire, on one of his assistants, and the action was that of a powerful narcotic. The young man stated that he felt all the symptoms of intoxication.

Dr. Ure had been recommended the use of the extract of hemp by his son ; but although he tried it for some time, he never experienced the slightest effect from it. The extract was the same as the above, having been obtained from Mr. Squire.

The Chairman thought that the present state of medical knowledge, in reference to the action of Indian hemp, was very unsatisfactory and imperfect.—*Dublin Med. Press.*

